# Inter-Cellular Exchange of Cellular Components via VE-Cadherin-Dependent Trans-Endocytosis

**DOI:** 10.1371/journal.pone.0090736

**Published:** 2014-03-06

**Authors:** Takashi Sakurai, Melissa J. Woolls, Suk-Won Jin, Masahiro Murakami, Michael Simons

**Affiliations:** 1 Yale Cardiovascular Research Center and Section of Cardiovascular Medicine, Department of Internal Medicine, Yale University School of Medicine, New Haven, Connecticut, United States of America; 2 Department of Cell Biology, Yale University School of Medicine, New Haven, Connecticut, United States of America; University of Illinois at Chicago, United States of America

## Abstract

Cell-cell communications typically involve receptor-mediated signaling initiated by soluble or cell-bound ligands. Here, we report a unique mode of endocytosis: proteins originating from cell-cell junctions and cytosolic cellular components from the neighboring cell are internalized, leading to direct exchange of cellular components between two adjacent endothelial cells. VE-cadherins form transcellular bridges between two endothelial cells that are the basis of adherence junctions. At such adherens junction sites, we observed the movement of the entire VE-cadherin molecule from one endothelial cell into the other with junctional and cytoplasmic components. This phenomenon, here termed trans-endocytosis, requires the establishment of a VE-cadherin homodimer *in trans* with internalization proceeding in a Rac1-, and actomyosin-dependent manner. Importantly, the trans-endocytosis is not dependent on any known endocytic pathway including clathrin-dependent endocytosis, macropinocytosis or phagocytosis. This novel form of cell-cell communications, leading to a direct exchange of cellular components, was observed in 2D and 3D-cultured endothelial cells as well as in the developing zebrafish vasculature.

## Introduction

Intercellular communications are critically important for fundamental functions of multicellular organisms including plants and humans. These signals are commonly mediated by transmembrane channels transferring small molecules or by receptors binding ligands, such as soluble, cell surface proteins, or extracellular matrix components. Endothelial and epithelial cells are unique with regard to their ability to form tight cell-cell adhesion structures which are dynamically remodeled during tissue morphogenesis. These structures also play an important role in regulation of a number of biological processes, including permeability, cell trafficking, and signal transduction from soluble proteins and extracellular matrix components. The main adhesive structure of endothelial junctions is the vascular endothelial cadherin (VEC)-based complex. This complex is capable of controlling a number of endothelial functions via VEC internalization, endocytic trafficking, and recycling [1], [2].

Among its many biological functions, VEC plays an important role in the maintenance of vascular integrity due to its involvement in the formation of adherens junction [3]. The VEC extracellular domain includes five cadherin repeats, with the most N-terminal repeat being critically involved in the formation of homophilic adhesions *in trans* with VEC expressed on neighboring cells [4], [5]. The intracellular domain of VEC forms protein complexes with a number of cytoplasmic proteins, including β-catenin and p120 catenin, that can then bind to α-catenin, linking VE-cadherin to the actin cytoskeleton [1], [6].

As with most transmembrane proteins, cadherins have been reported to be internalized and recycled to the plasma membrane [7]–[9]. In the process of studying VEC endocytosis using fluorescently tagged VEC examined by spinning disk confocal microscopy in live cells, we observed that VEC tagged proteins were sometimes observed in a different endothelial cell than those they were originally expressed in. Examination of this phenomenon uncovered an alternative mode of VEC trafficking, here termed trans-endocytosis, involving direct internalization of the VEC-VEC dimer bridging the two endothelial cells into one of the cells. The resulting vesicles contained not only the whole VEC molecule, but also VEC- associated proteins and even cytoplasmic components, including EGFP and siRNAs. Importantly, the process of VEC-dependent trans-endocytosis was not affected by the use of inhibitors of clathrin-dependent endocytosis, macropinocytosis or phagocytosis but did require actomyosin force generation and Rac1 activity.

## Results

We first employed the COS7 cell system commonly used for analyzing the function of ectopically expressed VEC. We transduced cells with fluorescently tagged VEC by lentiviral expression system. COS7 cells normally use N-cadherin to form cell-cell junctions; however, forced expression of VEC excludes N-cadherin from junctions resulting in the formation of VEC-based cell-cell adhesion (Figure S1A). VEC-EGFP and VEC-TagRFPT were first transduced into separate cell populations. As the VEC-based junction is subject to dynamic remodeling with constitutive internalization and recycling, we observed abundant intracellular vesicles containing tagged VEC. In control cells expressing only VEC-EGFP or VEC-TagRFPT, there was almost no “opposite color” fluorescence detected, save for very slight auto-fluorescence in red or green channel, respectively (data not shown). However, mixing of the two populations of COS7 cells expressing either VEC-EGFP [10] or VEC-TagRFPT created a cell-cell interface consisting of a mixture of VEC-EGFP and VEC-TagRFPT (data not shown). When the two populations of COS7 cells expressing either VEC-EGFP or VEC-TagRFPT were co-cultured, we observed internalized VEC fused proteins of both flourophores: *i.e.* VEC-TagRFP molecules in VEC-EGFP expressing cells (data not shown). Because VEC can be cleaved at the membrane-cytoplasm interface [11], [12], EGFP fluorescent protein was fused to the C-terminus of VEC to permit tracking of the full length VEC molecule. VEC-EGFP was found in TagRFPT positive cells, and vice versa, indicating that the full length VEC was transferred from one cell to the adjacent cell.

**Figure 1:**
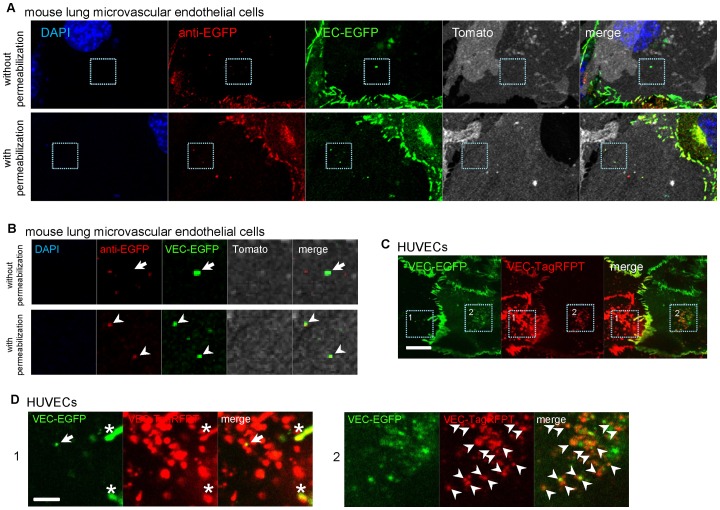
VEC molecules are internalized by adjacent cells via trans-endocytosis. (**A**) Co-culture of primary microvascular endothelial cells from mice lungs. The primary microvascular endothelial cells were isolated separately from VEC-EGFP knock-in mice and Rosa26-mTmG mice expressing mTomato fluorescent protein in all cells and fixed after 24 hours of the co-culture. The trans-endocytosed VEC-EGFP molecules in mTomato fluorescent protein expressing cells were stained by anti-GFP antibody without (upper images) and with permeabilization (lower images). (**B**) Higher magnification of images of the indicated area in A. Arrows in upper images show VEC-EGFP molecule could not be stained by anti-GFP antibody in non-permeabilized cells. Arrowheads in lower images show VEC-EGFP molecule stained by anti-GFP antibody in permeabilized cells. (**C**) Co-culture of HUVECs expressing VEC-EGFP and HUVECs expressing VEC-TagRFPT. Trans-endocytosis of VEC occurred in HUVECs (scale bar  = 20 µm). (**D**) Higher magnification of the indicated area in C. Arrow shows VEC-EGFP molecules were trans-endocytosed by VEC-TagRFPT expressing cells. Asterisks show the extended filopodia or adherens junctions from the neighboring cell. Arrow heads show VEC-TagRFPT molecules were trans-endocytosed by VEC-EGFP expressing cells. Scale bar  = 5 µm.

The same VEC trans-endocytosis was observed in isolated primary microvascular endothelial cells from mice (Figure 1A and 1B). To prove that these trans-endocytosed VEC-EGFP molecules were internalized by the cells, we performed immunostaining of fixed cells by anti-GFP antibody with/without permeabilization. Primary microvascular endothelial cells were isolated separately from VEC-EGFP knock-in mice and Rosa26-mTmG mice expressing mTomato fluorescent protein in all cells and fixed after 24 hours of the co-culture. Proteins inside of non-permeabilized cells would not be stained by antibodies [13]. The fusion protein, VEC-EGFP, can be detected by 488 nm laser excitation without permeabilization. If VEC-EGFP molecules are trans-endocytosed by Rosa26-mTmG endothelial cells and then internalized, VEC-EGFP in Rosa26-mTmG endothelial cells will be detected by anti-GFP antibody only in permeabilized cells, but not in cells without permeabilization. The results of the immunostaining showed that the VEC-EGFP molecules were detectable in the EGFP channel and could not be stained by anti-GFP antibody when cells had not been permeabilized before immunostaining (Arrows in Figure 1B, upper panel). However, the VEC-EGFP molecules were clearly stained by anti-GFP antibody when cells had been permeabilized (Arrow heads in Figure 1B, lower panel). These results suggest these trans-endocytosed VEC-EGFP molecules are inside of cells.

Furthermore, when VEC-EGFP and VEC-TagRFPT were transduced by lentivirus in human umbilical venous endothelial cells (HUVECs), the trans-endocytosis of VEC was also observed (Figure 1C and 1D). And when cells were transduced with VEC with small tag, VEC-FLAG and VEC-HA, the trans-endocytosis of VEC was also observed (unpublished data).

To study the kinetics of this process, two sets of cells, COS7 cells expressing either VEC-EGFP or VEC-TagRFPT were mixed together. Then we tracked the same cell over time and measured the number of trans-endocytosed VEC-TagRFPT molecules in the EGFP positive cell. There was a gradual increase of trans-endocytosed molecules over time, starting at 1 hr after formation of cell-cell contacts that reached plateau by 3 hours (Figure S1B and S1C). When repeated in HUVECs cells, the kinetics were similar (unpublished data).

To exclude the possibility that a fluorescent signal is arising from cellular auto-fluorescence or bleed-through originating from fluorescent cross-talk, we made a VEC fused protein using mKikGR fluorescent protein. Fluorescence of mKikGR can be irreversibly converted from green to red by photo-activation using UV or violet light (350–410 nm) [14], [15]. When the two populations of HUVECs expressing VEC-EGFP and VEC-mKikGR were co-cultured, there was almost no red fluorescence detected before photo-activation (Figure S1D). After photo-activation, however, we observed red VEC-mKikGR molecules in VEC-EGFP expressing cells (Figure S1E). The red fluorescence observed is not from auto-fluorescence, because they were not detected before photo-activation. Regardless of the extent of activation of VEC-mKikGR, enhanced red fluorescence in VEC-EGFP expressing cells meant the presence of VEC-mKikGR molecule in the VEC-EGFP expressing cells. This result clearly shows the red fluorescence was not due to auto-fluorescence, but due to the presence of trans-endocytosed whole VEC-mKikGR molecules from neighboring cells. When HUVECs expressing VEC-EGFP and VEC-mKikGR in the normal growth medium were mixed together, there was a gradual increase in the number cells demonstrating the presence of trans-endocytosis molecules over time reaching over 80%, by 8 hours (Figure S1F and S1G). Virtually all cells that had cell-cell contact with neighboring cells demonstrated trans-endocytosis of VEC with a frequency of over 80%, 10 h after plating.

**Figure 2:**
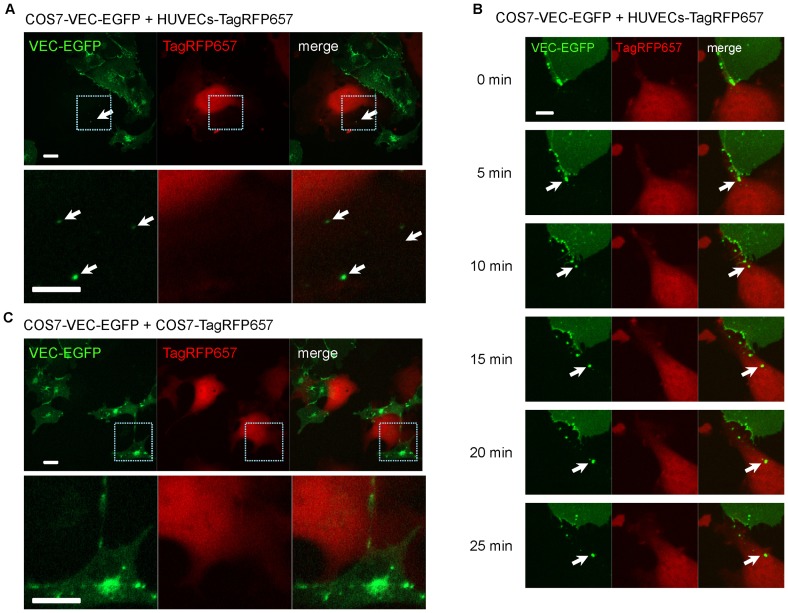
VEC trans-endocytosis occurs between HUVECs expressing endogenous VEC and COS7 cells expressing VEC fusion proteins. (**A**) Co-culture of COS7 cells expressing VEC-EGFP (COS7-VEC-EGFP) and HUVECs expressing TagRFP657 (HUVEC-TagRFP657). Lower images are higher magnification of the indicated area in upper images. VEC trans-endocytosis occurred between COS7-VEC-EGFP and HUVEC-TagRFP657, due to endogenous VEC in HUVECs. Exogenously expressed VEC molecules in COS7 cells form cell-cell junctions. Arrows show trans-endocytosed VEC-EGFP molecules from COS7 cells by adjacent HUVECs labeled with TagRFP657. Scale bars  = 20 µm. (**B**) Time-course images of co-culture of COS7-VEC-EGFP and HUVECs-VEC-TagRFP657. The interaction between endogenous VEC in HUVECs and over-expressed VEC-EGFP in COS7 cells can induce trans-endocytosis of VEC. Arrows show the trans-endocytosed VEC-EGFP molecules from a COS7 cell that appeared to bud off from cell-cell junctions and be pulled into a HUVEC. Scale bar  = 10 µm. See also Movie S1. (**C**) Co-culture of COS7 cells expressing VEC-EGFP (COS7-VEC-EGFP) and COS7 cells expressing TagRFP657 (COS7-TagRFP657). Lower images are higher magnification of the indicated area in upper images. No trans-endocytosis occurred between these cells. Scale bars  = 20 µm.

Because fluorescent proteins were fused to the carboxyl-terminus of VEC, which is located in the cytosol, these results suggest that the entire VEC molecule containing the fluorescent protein is internalized, presumably via homophilic extracellular VEC-VEC interaction. To demonstrate that this indeed is the case, we expressed only one set of VEC constructs (either VEC-EGFP or VEC-TagRFPT) in COS7 cells that do not normally express VEC, and co-cultured these COS7 cells with HUVECs that express endogenous VEC. When we mixed COS7 cells expressing VEC-EGFP with HUVECs, we could observe trans-endocytosis between an endogenous VEC in HUVECs and an over-expressed VEC-EGFP in COS7 cells (Figure 2A and 2B and Movie S1). The trans-endocytosed VEC-EGFP molecules from a COS7 cell appeared to bud off from cell-cell junctions and be pulled into a HUVEC (Figure 2B and Movie S1). No trans-endocytosis was detectable when COS7 cells expressing VEC-EGFP were mixed with COS7 cells that did not express endogenous VEC (Figure 2C). These results demonstrate that VEC-VEC homophilic interaction is required for trans-endocytosis.

Although trans-endocytosis appears to occur at cell-cell contacts, it is possible that the VEC complex is released as an exosome and is then incorporated into other cells by vesicular uptake [16], [17]. To evaluate this possibility we first cultured VEC-EGFP- or VEC-TagRFPT-transduced HUVECs on different plates and exchanged the conditioned media. However, no trans-endocytosis was observed under these conditions (unpublished data). Next, we utilized the transwell plates where VEC-EGFP-transduced HUVECs were seeded on the upper chamber and VEC-TagRFPT-transduced HUVECs were seeded on the lower chamber. Serial observations after plating showed no evidence of trans-endocytosis (Figure S2A), whereas the control plate in which these cells are mixed and plated clearly showed trans-endocytosed molecules. Additionally, media was fractionated for the exosomal fraction. This faction, while positive for the exosomal marker Syntenin, did not contain detectable VEC (Figure S2B). Finally, we disrupted cell-cell junction formation by introducing a point mutation in the VEC extracellular domain required for homophilic VEC-VEC binding [18]. Transduction of HUVECs with mutated VEC (VEC-W49A-TagRFPT) completely eliminated trans-endocytosis (Figure S2C). Taken together, these experiments demonstrate that trans-endocytosis occurs at VEC-formed cell-cell junction and is not driven by an exosome-mediated mechanism, but instead requires hemophilic VEC interactions.

**Figure 3:**
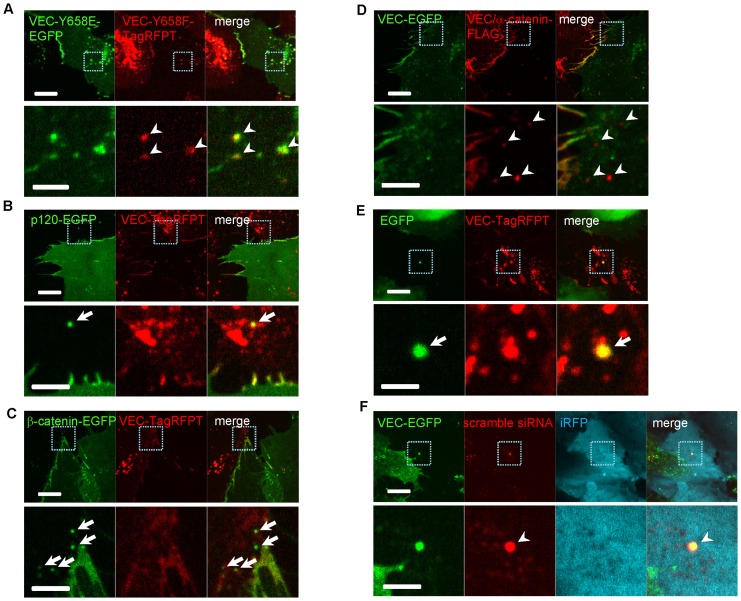
VEC trans-endocytosis mediates transport of junctional proteins. (**A**) Co-culture of HUVECs expressing VEC-Y658E-EGFP and HUVECs expressing VEC-Y658F-TagRFPT. Arrow heads show VEC-Y658F-TagRFPT molecules were trans-endocytosed by VEC-Y658E-EGFP expressing cells. (**B**) Co-culture of HUVECs expressing p120-EGFP and HUVECs expressing VEC-TagRFPT. Arrow shows p120-EGFP molecules were trans-endocytosed by VEC-TagRFPT expressing cells. (**C**) Co-culture of HUVECs expressing β-catenin-EGFP and HUVECs expressing VEC-TagRFPT. Arrows show β-catenin -EGFP molecules were trans-endocytosed by VEC-TagRFPT expressing cells. (**D**) Co-culture of HUVECs expressing VEC-EGFP and HUVECs expressing VEC/α-catenin-FLAG. Arrowheads show VEC/α-catenin-FLAG molecules were trans-endocytosed by VEC-EGFP expressing cells. (**E**) Co-culture of HUVECs expressing EGFP and HUVECs expressing VEC-TagRFPT. Arrow shows EGFP molecules were trans-endocytosed and co-localized with VEC-TagRFPT in the recipient cell. (**F**) Co-culture of HUVECs labeled with iRFP and VEC-EGFP expressing HUVECs transfected with Cy3-labeled scramble siRNA. Arrowhead shows siRNA molecules were trans-endocytosed and co-localized with VEC-EGFP in the recipient cell. (**A–F**) Lower images are higher magnification of the indicated area in upper images. Scale bars  = 20 µm, upper images; 5 µm, lower images.

To further define molecular events regulating trans-endocytosis, we examined the role of one VEC-binding protein, p120-catenin. A VEC mutant with a disrupted p120-catenin binding site (VEC-Y658E), and another mutant VEC-Y658F, which constitutively binds with p120 [10], [19], were used for these studies. Trans-endocytosis was observed when both VEC-Y658E and VEC-Y658F mutants were expressed in HUVEC (Figure 3A). Moreover, p120-catenin was also detected in the trans-endocytosed proteins (Figure 3B), suggesting that the mechanism differs from the conventional VEC internalization, which requires VEC-p120 dissociation [8]. Additionally, another cytosolic VEC interacting protein, β-catenin, was also trans-endocytosed by adjacent cells (Figure 3C). Furthermore, β-catenin- (Figure S3A) or p120-catenin-EGFP (Figure S3B) and VEC-TagRFPT were co-expressed in COS7 cells and co-cultured with HUVECs expressing iRFP, a far-red fluorescent protein. We found both p120- or β-catenin-EGFP and VEC-TagRFPT were trans-endocytosed by neighboring cells simultaneously.

We next set to examine the extent of the protein complex that is trans-endocytosed in this VEC-VEC interaction-driven process. To this end, we used a FLAG-tagged VEC fused to α-catenin (VEC/α-catenin-FLAG). Expression of this construct in HUVECs leads to formation of highly stabilized adherens junctions [20]. Nevertheless, the entire VEC/α-catenin-FLAG construct was observed to be trans-endocytosed by adjacent cells (Figure 3D). Furthermore, cytosolic components such as EGFP were also trans-endocytosed by adjacent cells (Figure 3E). When HUVECs expressing EGFP were co-cultured with HUVECs expressing VEC-TagRFPT, EGFP was detected in VEC-TagRFPT expressing cells (Figure 3E). The trans-endocytosis of cytosolic components, like EGFP molecule, suggests that genomic components like mRNA and miRNA also trans-endocytosed by adjacent cells which would potentially convert the property of the adjacent cells. We confirmed that transfected siRNA molecules were also trans-endocytosed by adjacent cells (Figure 3F). When HUVECs expressing iRFP were co-cultured with VEC-EGFP expressing HUVECs transfected with scramble siRNA labeled with Cy3, siRNA molecules were trans-endocytosed by iRFP expressing cells, and those siRNA molecules co-localized with VEC-EGFP (Figure 3F). These data suggest that not only the entire VEC molecules, but also a large part of the protein complex associated with VEC including cytosolic components was trans-endocytosed by adjacent cells.

**Figure 4:**
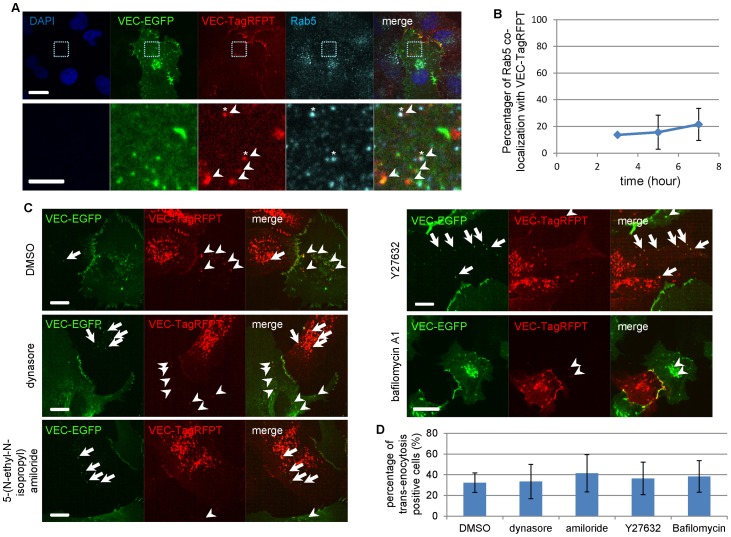
VEC trans-endocytosis is not dependent on known endocytic pathway. (**A**) Co-culture of HUVECs expressing VEC-EGFP and HUVECs expressing VEC-TagRFPT, then stained with anti-Rab5 antibody. The trans-endocytosed VEC molecules by the adjacent cell co-localized with Rab5 in the recipient cells to a low extent. Lower images are higher magnification of the indicated area in upper images. Scale bars  = 20 µm, upper images; 5 µm, lower images. (**B**) Quantitative analysis of the number of trans-endocytosed VEC molecules co-localized with Rab5 in the recipient cells. About 15–20% of trans-endocytosed VEC molecules co-localized with Rab5 in the recipient cells. The percentage of Rab5 co-localization with VEC-TagRFPT was counted over 6-9 different fields of view for each time point; n = 6 (3 h), n = 8 (5 h) and n = 10 (7 h). Data were expressed as mean ± SD. (**C**) Co-culture of COS7 cells expressing VEC-EGFP and COS7 cells expressing VEC-TagRFPT with or without various inhibitors. Arrows show the trans-endocytosed VEC-EGFP molecules by VEC-TagRFPT expressing cells. Arrowheads show trans-endocytosed VEC-TagRFPT molecules by VEC-EGFP expressing cells. The trans-endocytosis occurred even with several inhibitors for clathrin-dependent endocytosis, macropinocytosis or phagocytosis. Dynasore (20 µM), an inhibitor for clathrin/dynamin dependent endocytosis, 5-(N-ethyl-N-isopropyl) amiloride (25 µM), the macropinocytosis inhibitor, Y27632 (10 µM), ROCK inhibitor, and bafilomycin A1 (200 nM), a specific inhibitor of the vacuolar type H(+)-ATPase for phagocytosis were used. Scale bars  = 20 µm. (**D**) Quantification of the number of trans-endocytosis positive cells with indicated inhibitors. The percentage of trans-endocytosis positive cells was counted over 10 different fields of view for each inhibitor; n = 88 (DMSO), n = 65 (dynasore), n = 57 (amiloride), n = 63 (Y27632) and n = 57 (bafilomycin A1). Data were expressed as mean ± SD.

We next examined whether the VEC complex trans-endocytosis involved one of the standard endocytic pathways. The trans-endocytosed VEC from the neighboring cell did not co-localize with internalized transferrin (Figure S4A), while a small subset of Rab5-positive endosomes in the recipient cells co-localized with trans-endocytosed VEC (Figure 4A and 4B). This suggests that the VEC trans-endocytosis is occurring via an unconventional clathrin-independent pathway, and that some of the internalized VEC-containing structures may be merging with the Rab5 pathway [21] after clathrin-independent internalization. We carried out co-culture of VEC-EGFP-expressing HUVECs with mRFP-Rab5 or mRFP-Rab5-DN expressing cells and found that trans-endocytosis was not inhibited by Rab5-DN expression (Figure S4B). These results suggest Rab5 is not involved in the process of VEC trans-endocytosis.

Treatment with either dynasore (20 µM), an inhibitor of clathrin/dynamin dependent endocytosis, 5-(N-ethyl-N-isopropyl) amiloride (25 µM), an inhibitor of macropinocytosis and exosome uptake, bafilomycin A1 (200 nM), a specific inhibitor of vacuolar type H(+)-ATPase for phagocytosis, or Y27632, Rock inhibitor [22], did not inhibit the trans-endocytosis (Figure 4C and 4D). Additionally, none of the siRNAs against proteins involved in macropinocytosis, including ARF1 [23], ARF6 [24], ANKFY1 [25] and CtBP [26], had any effect on trans-endocytosis (Figure S4C). These results suggest that the trans-endocytosis is not dependent on any known endocytic pathway including clathrin-dependent endocytosis, macropinocytosis or phagocytosis.

The internalized complexes containing the VEC molecule from the neighboring cell co-localized with a subset of Rab7-positive endosomes and a small subset of Rab5- and Rab11-positive endosomes, in the receiving cells (Figure S5). This suggests that trans-endocytosed complexes behave in a way similar to endosomes and may cycle back to the membrane or be degraded.

**Figure 5:**
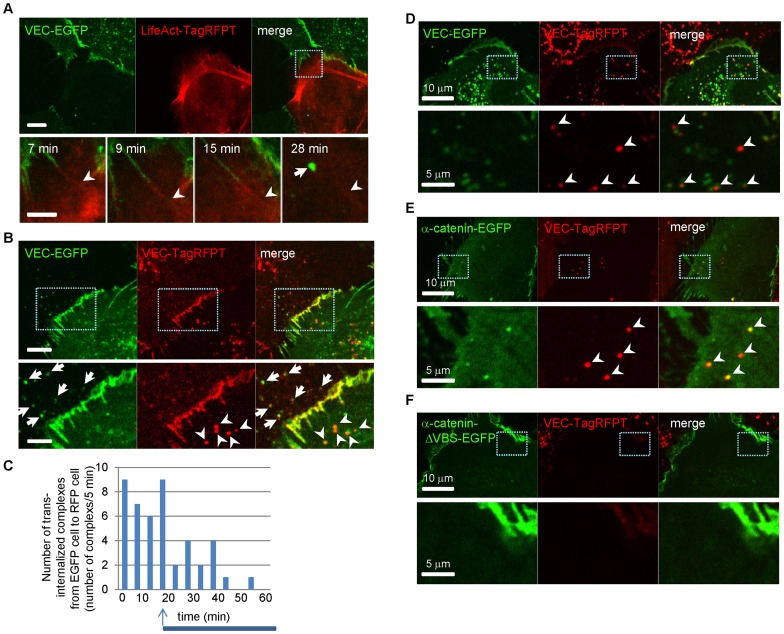
VEC trans-endocytosis is dependent on actin/myosin force and Vinculin. (**A**) Co-culture of HUVECs expressing VEC-EGFP and HUVECs expressing LifeAct-TagRFPT. The trans-endocytosed VEC-EGFP structure clearly associated with F-actin visualized by LifeAct-TagRFPT. Lower images are time-course images of higher magnification of the indicated area in upper images (only for merged image). Arrow shows the trans-endocytosed VEC-EGFP structure and arrowheads show F-actin associated with VEC-EGFP during the trans-endocytosis process. Scale bars  = 10 µm, upper images; 5 µm, lower images. See also Movie S2. (**B**) Co-culture of HUVECs expressing VEC-EGFP and HUVECs expressing VEC-TagRFPT with (−)-blebbistatin. (−)-Blebbistatin, a selective inhibitor of non-muscle myosin II, inhibited trans-endocytosis of VEC vesicles. Arrows show trans-endocytosed VEC-EGFP vesicles by VEC-TagRFPT expressing cells. Scale bars  = 10 µm, upper images; 5 µm, lower images. See also Movie S3. (**C**) Quantitative analysis of the number of trans-endocytosed VEC-EGFP structures from VEC-EGFP expressing cells to VEC-TagRFPT expressing cells in B. The number of trans-endocytosed vesicles after 5 minutes was counted and plotted. 100 µM of (−)-blebbistatin were added at 20 minutes after starting the time-lapse acquisition to inhibit the trans-endocytosis of VEC. (**D–F**) Co-culture of HUVECs expressing VEC-EGFP or α-catenin constructs and HUVECs expressing VEC-TagRFPT. When HUVECs expressing VEC-TagRFPT were co-cultured with HUVECs expressing VEC-EGFP (D) or α-catenin-EGFP (E), trans-endocytosis of VEC occurred as shown by arrowheads. When HUVECs expressing VEC-TagRFPT were co-cultured with HUVECs expressing α-catenin-ΔVBS-EGFP (F), trans-endocytosis of VEC did not occur. Lower images are higher magnification of the indicated area in upper images. Scale bars  = 10 µm, upper images; 5 µm, lower images.

To determine if trans-endocytosis is driven by contractile force generated by the actomyosin cytoskeleton, we used LifeAct polypeptide [27] to visualize F-actin bundles in living cells. Co-culture of HUVECs expressing VEC-EGFP and LifeAct-TagRFPT showed a clear association between VEC positive trans-endocytosed structures with F-actin fibers (Figure 5A and Movie S2). Treatment with (−)-blebbistatin, a selective inhibitor of non-muscle myosin II [28], [29], inhibited trans-endocytosis of VEC (Figure 5B, 5C and Movie S3). There were many trans-endocytosed molecules moving in both directions, *i.e.*, VEC-EGFP molecules were trans-endocytosed by the RFP-positive cell and VEC-TagRFPT molecules were trans-endocytosed by the EGFP-positive cells, before adding (−)-blebbistatin at 20 minutes of time-point in the Movie S3. However, after adding (−)-blebbistatin, the movement decreased dramatically (Figure 5C and Movie S3). These data suggest that trans-endocytosis is driven by actomyosin contractility.

Vinculin is thought to be involved in the transfer of actomyosin contractile force to cadherin-complex [30], [31]. To evaluate vinculin involvement in trans-endocytosis, we over-expressed α-catenin constructs with mutations in the vinculin binding site (VBS) (α-catenin-ΔVBS-EGFP) [30]. Overexpression of α-catenin-ΔVBS-EGFP precludes binding of endogenous vinculin to the VEC-complex [30]. Trans-endocytosis of VEC occurred when native α-catenin or α-catenin-EGFP was expressed in HUVECs, but not when α-catenin-ΔVBS-EGFP was over-expressed in HUVECs (Figure 5D, 5E and 5F). These results suggest that actin-mediated pulling force on the VEC complex is involved in the trans-endocytosis process.

**Figure 6:**
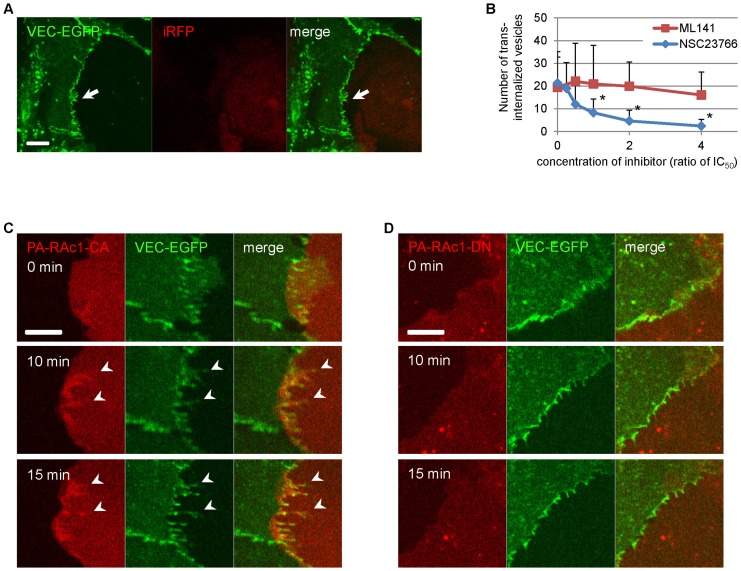
VEC trans-endocytosis is Rac1 dependent. (**A**) Co-culture of HUVECs expressing VEC-EGFP and HUVECs expressing iRFP with 100 µM of NSC23766. NSC23766, a specific inhibitor for Rac1 activation, inhibited trans-endocytosis of VEC-EGFP, though cell-cell junctions remained intact. Arrow shows intact cell-cell junction. Cells were first pre-treated for an hour with 100 µM of NSC23766 before co-culture, then were mixed and incubated for 4 hours with 100 µM of NSC23766. Scale bar  = 10 µm. (**B**) Quantitative analysis of the number of trans-endocytosed structures with Rac1 or Cdc42 inhibitors. ML141, a specific inhibitor of Cdc42, and NSC23766 were used at concentrations around their IC_50_ values. The IC_50_ values of ML141 and NSC23766 are 2.6 µM and 50 µM, respectively. NSC23766 inhibited trans-endocytosis of VEC in a dose-dependent manner. The number of trans-endocytosed structures was counted for over 10-13 different fields of view per time point; n = 24-35 (ML141) and n = 19-38 (NSC23766). *, p<0.01 vs DMSO. Data were expressed as mean ± SD. (**C**) Co-culture of HUVECs expressing PA-Rac1-CA and HUVECs expressing VEC-EGFP. PA-Rac1-CA was accumulated at cell-cell junctions and co-localized with VEC-EGFP after its activation at 0 time point. Arrowheads show co-localization of PA-Rac1-CA with VEC-EGFP at cell-cell junctions. Scale bar  = 10 µm. See also Movie S4. (**D**) Co-culture of HUVECs expressing PA-Rac1-DN and HUVECs expressing VEC-EGFP. PA-Rac1-DN was not accumulated at cell-cell junctions, nor co-localized with VEC-EGFP after its activation at 0 time point. Scale bar  = 10 µm. See also Movie S4.

Further studies revealed that treatment with NSC23766, a specific inhibitor of Rac1 that targets its activation by guanine nucleotide exchange factors, (GEFs), Tiam1 and TrinoN [32], inhibited the trans-endocytosis in a dose-dependent manner, whereas ML141, an inhibitor of cdc42 Rho family GTPase, had no effect (Figure 6A and 6B). Of note, adherens junctions were not disturbed by NSC23766 treatment (Figure 6A). Another Rac1 inhibitor, W56, also inhibited the VEC trans-endocytosis in a time-dependent manner (Figure S6). These results suggest that VEC-driven trans-endocytosis is a Rac1-dependent process.

In order to further define the role of Rac1 in VEC trans-endocytosis, we used photo-activatable (PA) Rac1 constructs, which were fused with the photoreactive domain from phototropin. These constructs block Rac1 interactions until photo-activation [33]. Co-culture of HUVECs expressing PA- constitutively active Rac1 (Rac1-CA) and VEC-EGFP revealed that PA-Rac1-CA accumulated at cell-cell junction and co-localized with VEC-EGFP after its activation, while PA-dominant negative Rac1 (Rac1-DN) did not (Figure 6C and 6D, Movie S4). This further demonstrates that Rac1 activation is directly involved in VEC trans-endocytosis at the cell-cell junction.

**Figure 7:**
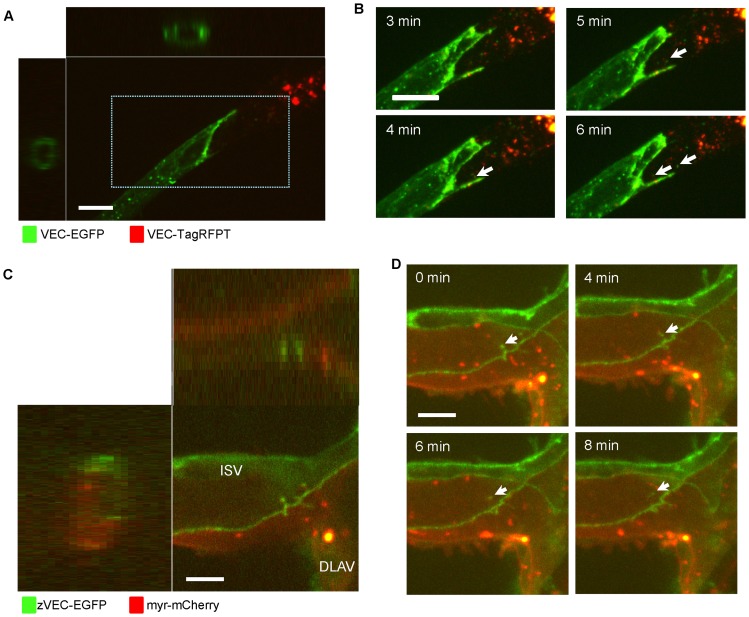
VEC trans-endocytosis occurs in sprouting HUVECs and endothelial cells in zebrafish embryos. (**A**) A single z-plane, x-z and y-z cross-sectional images of the tube-like structure of sprouting HUVECs. HUVECs expressing VEC-EGFP and HUVECs expressing VEC-TagRFPT were co-cultured in three-dimension fibrin gels. The Z-stack images were taken at the connection between HUVECs expressing VEC-EGFP and HUVECs expressing VEC-TagRFPT. Images were collected at 0.3 µm intervals with the 488 nm and 561 nm lasers to create a stack in the Z axis with a 60x objective. Scale bar  = 20 µm. See also Movie S5. (**B**) Higher magnification of a 3-dimensional projection image of the indicated area in A. Arrows show VEC-EGFP molecules were trans-endocytosed by HUVECs expressing VEC-TagRFPT. Scale bar  = 10 µm. (**C**) A three dimensional projection image, x-z and y-z cross-sectional images of the connection between the dorsal longitudinal anastomotic vessel (DLAV) and an intersegmental vessel (ISV) of a zebrafish embryo. Zebrafish VEC-EGFP (zVEC-EGFP) plasmids were injected into Tg(flkl:myr-mCherry) zebrafish using Tol2 system for transient mosaic expression of zVEC-EGFP. Images were collected at 1 µm intervals using the 488 nm and 561 nm lasers to create a stack in the Z axis with a 60x objective. Scale bar  = 5 µm. See also Movie S6. (**D**) Sequential 3-dimensional projection image of the zebrafish vessel in C. Arrows show a zVEC-EGFP positive structure budding to inside of the endothelial cell. Scale bars  = 5 µm.

Finally, we confirmed that the existence of trans-endocytosis occurs in a three-dimensional culture using a HUVEC sprouting assay, and in vivo, using live zebrafish embryos (Figure 7). Co-culture of HUVECs expressing VEC-EGFP and VEC-TagRFPT, in a three-dimensional fibrin gel culture showed VEC-EGFP molecules were trans-endocytosed by VEC-TagRFPT expressing HUVECs, forming tube-like structures in the fibrin gel (Figure 7A and 7B, Movie S5).

Zebrafish VEC-EGFP (zVEC-EGFP) plasmids were injected into Tg(flkl:myr-mCherry) zebrafish using the Tol2 system to induce transient mosaic expression of zVEC-EGFP. At the one cell stage, VEC-EGFP plasmids were injected into Tg(flkl:myr-mCherry) zebrafish, which express ras-mCherry in all endothelial cells. For time lapse analysis, embryos were placed in 3.5 cm glass bottom dishes in egg water with 0.016% tricaine to prevent movement. Zebrafish VEC-EGFP endothelial expression was analyzed at 30–80 hr post fertilization using spinning disk microscopy focusing on the connection between the dorsal longitudinal anastomotic vessel (DLAV) and an intersegmental vessel (ISV), where was the best region to observe vessels to detect signals from VEC-EGFP. Time-lapse images demonstrated a VEC-EGFP positive structure budding to adjacent endothelial cells (Figure 7C and 7D, Movie S6). This is similar to the manner in which VEC-EGFP molecules were trans-endocytosed by adjacent cells in cultured cells (Figure 2B).

## Discussion

**Figure 8:**
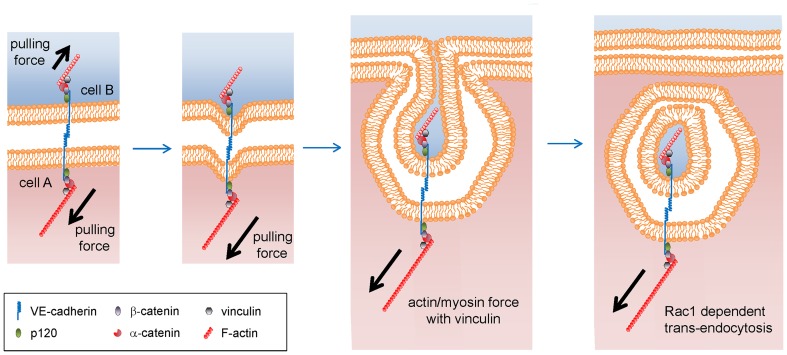
Model of trans-endocytosis of VEC. VEC complex, VEC, p120, β-catenin, α-catenin and vinculin are pulled into the cell by actin/myosin force in a Rac1-dependent manner. The trans-endocytosed structure also contains some volume of adjacent cytosolic components that may include proteins or RNA molecules.

To our knowledge, our results show a new paradigm of cellular communication that achieves a direct transfer of cellular materials between adjacent cells via cell-cell adhesion-dependent trans-endocytosis. VEC expression and homophilic interactions are necessary for this process, which depends on formation of a VEC bridge. Two cells making cell-cell contacts are pulling each other by actomyosin force via the VEC bridges. When the force from one of the cell exceeds that of the other cell, the cell internalizes the VEC complex with its partner in an actin/myosin- and Rac1-dependent manner. The model of VEC-driven trans-endocytosis is summarized in Figure 8.

The best way to provide further evidence of the VEC trans-endocytosis is to carry out a thorough EM analysis. This will provide strong evidence for this model and is the scope of future investigation. To further clarify the precise mechanism of trans-endocytosis, it is of particular interest to understand whether trans-endocytosis requires tension across VEC. It has been reported that fluid shear stress reduces the tension across VEC adhesion [34]. It would be interesting to demonstrate the relationship between tension across VE-cadherin adhesion and the rate of trans-endocytosis under physiological condition. Also, the distribution of pulling and pushing forces at the site of trans-endocytosis needs to be clarified. Using a validated FRET-based VEC tension sensor [34] will address this question.

We first observed “trans-endocytosis” in confluent endothelial monolayers and then demonstrated that it also occurred when endothelial cells are subconfluent using time-lapse imaging. It is very difficult to distinguish trans-endocytosis occurring while cells are subconfluent vs. the same process taking place in the confluent monolayer since fluorescent molecule trans-endocytosed during subconfluent period remain in the cells after the confluent monolayer forms. Distinguishing these two possibilities would require development of a more sophisticated approach. Most of data in the manuscript has been done in the subconfluent conditions. Further experiments to clarify whether “trans-endocytosis” occurs in contiguous monolayers would require the use of SNAP- [35] and/or CLIP-tags [36] to label membrane VE-cadherin molecule only after endothelial cells become confluent.

We often observed that the trans-endocytosed proteins in the recipient cell exclusively contained the fluorescence arising from the VEC-fusion protein of the neighboring cell and not the one expressed by the recipient cell, and *vice versa*. We speculate that this is attributed to the chemical property of fluorescent proteins known to be affected by pH. After molecules enter the endocytic process, they are trafficked to early endosomes and subsequently late endosomes followed by lysosomes. The pH in the vesicles decreases as they progress to the later stage, from about pH 6.8-5.9 in early endosomes to about pH 6.0-4.9 in late endosomes and lysosomes [37], [38]. GFP is acid-sensitive, pKa  = 5.8 [39], and TagRFP is highly acid-insensitive, pKa <4.0 [40]. Since most of our observations were done in live cell settings, the intensity of a fluorescent tagged protein might have been variably affected, with the highest susceptibility in EGFP, depending on the pH environment in their vesicles. Another explanation for this unbalanced fluorescence is the presence of endogenous VEC that occurred when we used the HUVEC system. The formation of adherens junctions between fluorescent tagged VEC and endogenous native VEC resulted in the fluorescence of only one color.

To study the role of Rac1, we first tried over-expression of Rac1-CA and Rac1-DN constructs. The expression of either resulted in a major change in cell morphology, rendering trans-endocytosis studies nearly impossible. To avoid that problem, we used the photo-activatable form of Rac1 constructs, namely PA-Rac1-CA and PA-Rac1-DN (Figure 6C and 6D). The benefit of PA-Rac1 constructs are that they act as constitutively or dominant negative form of Rac1 only when they are photo-activated by 488 nm laser. These results using PA-Rac1 constructs in Figure 6C and 6D clearly showed the role of Rac1 in trans-endocytosis. Because the use of Rac1 siRNA would result in the same morphology change problems that were observed with Rac1DN, we did not use Rac1 siRNA for the Rac1 signal depletion experiment.

The VEC trans-endocytosed structures may have a similar structure to the annular gap junction, with double bilayer lipid membranes containing some cytosolic components [41], [42]. The exchange of cellular components may play a role as a primitive form of cell-cell communication similar to the process by which *Escherichia coli* exchange their sex factor with transmission of F-factor plasmid by cell-cell contact [43].

Various kinds of processes similar to “trans-endocytosis” have been reported. Most are mediated by ligand/receptor interaction, such as EphrinB-Eph receptor [44], Notch- DLL ligand [45], [46], CD47-SHPS-1 ligand [47], and CD86-CTLA-4 [48]. All of these events are due to binding between different proteins. In distinction, VEC-driven trans-endocytosis is due to homophilic binding between two molecules of the same protein *in trans*. This suggests that cadherins are important for the cell-cell communication between the same types of cells expressing the same type of cadherins, which are conventionally considered to be important for the segregation of different cell populations. Similar to our finding, fragments of membranes called argosomes are transported over large distances intercellularly in the Drosophila wing disk to form morphogen gradients [49]. Also, in epithelial cells, claudin, a component of tight junctions, is co-endocytosed into adjacent cells while other components of tight junctions, such as JAM and ZO-1, are not co-endocytosed with claudin [50]. Recently, β-integrin-dependent transfer of neutrophil myeloperoxidase from neutrophils to ECs has been reported [51].

*In trans* movement of cellular components in this type of communication has also been reported in the transfer of double-membrane structures via very small spines, called spinules, in the adult rat hippocampus when studied by electron microscopy [52]. Tunneling of nano-tube like structures in immune cells [53], [54] and epithelial cells [55], the cellular bridge in neural crest cells [56], and direct cell-cell contact dependent intercellular transfer of cellular components are other reported examples of similar *in trans* cellular communication [57]. However, none of these have identified the mechanism responsible for the observed effects. The mechanism described here underlying VEC-dependent trans-endocytosis may be the driving force behind these observations perhaps utilizing VEC-related molecules such as N- or E-cadherins.

Using this mechanism, neighboring cells can exchange cellular material, including genetic material such as miRNAs, a function previously attributed only to gap junctions [58]-[60] and exosomes [61], [62]. The vesicles that are internalized from adjacent cells and have double bilayer lipid membranes may merge into an endosome, allowing the release of cytoplasmic components from neighboring cells to the recipient cell. While the full biological importance of this novel mode of cell-cell communication remains to be defined, the ability to exchange cellular components, exemplified by the transfer of cytosolic proteins (EGFP) and siRNA, argues for its potential significance.

## Experimental Procedures

### Ethics Statement

All animal experiments are performed accordingly to the protocol approved by the institutional animal care and use committee of Yale University.

### Cell culture and cells

Pooled human umbilical vein endothelial cells (HUVECs) from different donors (LONZA, Walkersville, MD, USA) were cultured in EBM-2 culture medium supplemented with EGM-2 bullet kit (LONZA) on gelatin-coated tissue dishes. HUVECs up to passage 6 were used for experiments. COS-7 cells, transformed African green monkey kidney fibroblast cells (ATCC, Manassas, VA, USA), and HEK293T cells (a gift from Dr. Yuanfei Wu, Yale University, New Haven, CT, USA) were cultured in Dulbecco's modified Eagle's medium (DMEM) (Invitrogen, Grand Island, NY, USA) supplemented with 10% fetal bovine serum and penicillin/streptomycin (Invitrogen). Primary mouse lung microvascular endothelial cells were isolated from mice lung by the method previously described [63], [64] and cultured in DMEM supplemented with 20% fetal bovine serum, penicillin/streptomycin, non-essential amino acids (Invitrogen) and 20 µg/ml endothelial mitogen (Biomedical Technologies, Inc., Stoughton, MA, USA).

### Antibodies and chemicals

Rabbit monoclonal anti-Rab5 antibody (3547) from Cell Signaling Technology (Danvers, MA, USA) was used at 1∶200 dilution for immunocytochemistry. Goat polyclonal anti-Syntenin antibody (ab53552) and rabbit polyclonal anti-calnexin antibody (ab137336) from abcam were used at 1∶100 dilution for immunocytochemistry. Rabbit polyclonal anti-GFP antibody (ab6556) from abcam was used at 1∶1,000 dilution for immunocytochemistry. Transferrin, Alexa Fluor 647 conjugate (T-23366) was purchased from Invitrogen. (−)-Blebbistatin (B0560) was purchased from Sigma (Sigma-Aldrich, St. Louis, MO, USA) and used at 100 µM. ML141 (71203-35-5) and NSC23766 (sc-204823) were purchased from R&D Systems (Minneapolis, MN, USA) and Santa Cruz Biotechnology (Dallas, TX, USA), respectively. AllStars negative control siRNA (1027281) was purchased from Qiagen (Hilden, Germany), and Silencer Cy3 Labeled GAPDH siRNA (AM4649) was purchased from invitrogen. Dynasore (D7693), 5-(N-ethyl-N-isopropyl) amiloride (A3085), Y27632 (Y0503), bafilomycin A1 (B1793) and W56 (W4392) were purchased from Sigma. siRNAs against ARF1, ARF6, ANKFY1 and CtBP were purchased from Santa Cruz Biotechnology and used at 12.5 nM final concentration with lipofectamine RNAiMAX (invitrogen).

### DNA constructs and viral transduction

Lentiviral transductions of HUVECs and COS-7 cells with human VEC fused to EGFP or TagRFP (a gift from Dr. Peter A. Vincent and Dr. Anthony M. Lowery, Albany Medical College, Albany, NY, USA) were performed using pLVX-IRES-Puro lentiviral expressing vector (Clontech, Mountain View, CA, USA) and Trono lab's third generation lentiviral packaging system (pMDL g/p RRE, pRSV-Rec and pMD2.G, Addgene plasmid 12251, 12253 and 12259, respectively). The serine residue at position 158 of TagRFP was substituted for tyrosine to make TagRFP-T (TagRFPT) to make a photo-stable and a higher fluorescence pKa mutant of TagRFP [65]. Photo-activatable fluorescent protein mKikGR (AM-V0150) were purchased from MBL international (Woburn, MA, USA). iRFP construct (plasmid 31857) was purchased from Addgene. VEC-Y658E and VEC-Y658F mutants were previously described [10]. TagRFP657 construct was a gift from Dr. Joerg Bewersdorf (Yale University). Mouse p120-3A-EGFP construct was a gift from Dr. Albert B. Reynolds (Vanderbilt University Medical Center, Nashville, TN, USA). Human β-catenin construct was a gift from Dr. Michael J. Caplan (Yale University). The constructs of EGFP-Rab5, mRFP-Rab5, mRFP-Rab5-DN, EGFP-Rab7 and EGFP-Rab11 were gifts from Dr. Yong Deng (Yale University). The sequence for LifeAct, a 17-amino-acid peptide (MGVADLIKKFESISKEE) [27] was synthesized and inserted into N-terminus of TagRFPT sequence in pLVX plasmid using XhoI and AgeI restriction enzymes. The trans-interaction deficient VEC mutant was generated by mutating the structurally important conserved tryptophan residue at position 49 in the first extracellular cadherin domain of VEC to alanine (VEC-W49A) [18]. Mouse α-catenin-EGFP and mouse α-catenin-ΔVBS-EGFP were a gift from Dr. Gerard van der Krogt (Hubrecht Institute, Utrecht, the Netherlands). PA-Rac1-constitutively active and PA-Rac1-dominant negative constructs were provided from Addgene (Addgene plasmid 22027 and 22029, respectively) [33]. Zebrafish VEC gene was cloned from zebrafish cDNA and cloned into pDestTol2pA2, with fli1 promoter sequence (Addgene, Plasmid 31160) and SV40 polyA signal, using Gateway system (Invitrogen).

### Immunofluorescence

Cells were washed with PBS, fixed with 2% paraformaldehyde (PFA) (18814, Polysciences, Inc, Warrington, PA, USA) in PBS for 10 minutes, permeabilized with 0.1% triton X-100 in PBS containing 2% PFA at room temperature for 5 minutes, and blocked with 3% bovine serum albumin (BSA) (IgG-Free, Protease-Free BSA (001-000-162), Jackson ImmunoResearch Laboratories, Inc., West Grove, PA, USA) at room temperature for 30 minutes. Cells were washed with PBS and incubated with diluted primary antibody at 4°C overnight, washed three times with PBS and incubated with diluted Alexa Fluor-conjugated secondary antibodies (1∶500 dilution) (Invitrogen) for 1 hour at room temperature. Then the slides were washed three times with PBS and mounted using Fluor-Gel (Electron Microscopy Sciences, Hatfield, PA, USA). Images were taken by Zeiss 510 laser scanning confocal microscopy, Zeiss 780 laser scanning confocal microscopy and PerkinElmer spinning disk microscopy confocal systems. Except images for Figure 7, images were taken at only one focal plane.

### Spinning disk live cell microscopy

For live cell imaging microscopy, cells were seeded on 3.5 cm glass bottom culture dishes (MatTek Corporation, Ashland, MA, USA) coated with 0.1% gelatin (G1393, Sigma). Time-lapse live cell imaging was performed with a PerkinElmer UltraView VoX spinning disk confocal system with a Nikon eclipse Ti inverted microscope and a Prior NanoScanZ 250 Micron motorized stage, enclosed by temperature controlled chamber with a CO_2_ environment system (IVS-3001) (In Vivo Scientific, LLC., St. Louis, MO, USA). The time-lapse images were acquired with a 60x oil NA 1.4 Plan Apochromat VC objective lens by using a chamber heater at 37°C. All images were enhanced for display with contrast adjustments in Volocity 6 software (PerkinElmer).

### Photo-activation of photo-activatable constructs

For photo-activation of mKikGR tagged protein, cells were photo-activated by 100% of 405 nm laser for 5 sec using PerkinElmer UltraView VoX spinning disk confocal system. For photo-activation of PA-Rac1 constructs, cells were photo-activated by 488 nm laser at the same laser power (10%) for other acquiring condition using PerkinElmer UltraView VoX spinning disk confocal system. Only photo-activated molecules in VEC-EGFP positive cells were counted in order to avoid counting auto-fluorescence in cells.

### Exosomal fractionation

To collect the exosomal fraction, we carried out fractionation using an ultracentrifuge (Sorvall MTX150, Thermo Scientific, Waltham, MA, USA) according to general methods for the exosomal fractionation [66]. Briefly, the culture medium, supplemented with exosomal free FBS, 24 hours after medium change was collected and centrifuged at 3,000×g for 10 min at 4°C. The supernatant was carefully transferred into a new tube and centrifuged at 10,000×g for 20 min at 4°C. The resulting supernatant was the carefully transferred into a new tube for ultracentrifuge and centrifuged at 100,000×g for 90 min at 4°C. The supernatant was carefully removed and pellet was re-suspended with sample buffer for western blotting and used as the exosomal fraction.

### siRNA transfer

HUVECs transduced with a lentiviral vector containing VEC-EGFP (HUVECs- VEC-EGFP) and without any lentiviral treatment (HUVECs-NT, non-treated) were maintained separately in 3.5 dishes. HUVECs-NT were transfected with Cy3 Labeled GAPDH siRNA according to Lipofectamine RNAiMax procedure. 24 hours after siRNA transfection, HUVECs- VEC-EGFP and HUVECs-NT were washed with PBS, trypsinized and mixed into 3.5 cm glass bottom dish. 4 hours after mix, the digital fluorescent images were captured by a PerkinElmer UltraView VoX spinning disk confocal system with a Nikon eclipse Ti inverted microscope.

### Inhibitor experiments

Two portions of HUVECs, expressing VEC-EGFP and VEC-TagRFPT cultured in 10 cm tissue culture dishes, were pre-treated with inhibitors (ML141, 1.25–10 µM; NSC23766, 12.5–200 µM) for an hour, then trypsinized and mixed into gelatin-coated glass bottom dishes. Cells were further incubated in a CO_2_ incubator at 37°C for 4 hour with inhibitors and analyzed using spinning disk microscopy.

### The three-dimensional fibrin gel culture of HUVECs

The three-dimensional culture experiments of HUVECs were performed using dextran-coated beads and a fibrin gel [67]. Two portions of HUVECs, expressing VEC-EGFP and VEC-TagRFPT cultured in 10 cm tissue culture dishes, were trypsinized and mixed with dextran-coated Cytodex3 microcarrier beads (Sigma) in 5 ml of EGM-2 MV media in 50 ml tube. Beads with cells were mixed by turning the tube upside down every 20 minutes for 3 hours at 37°C in 5% CO_2_ incubator. After incubation, beads with cells were transferred to a petri dish and incubated overnight at 37°C in 5% CO_2_ incubator. The following day, beads with cells were washed three times with EBM-2 supplemented with 2% FBS and re-suspended in 3 ml of EBM-2 supplemented with 2% FBS, 5 mg/ml fibrinogen (Sigma) and 50 µg/ml aprotinin (Sigma). Five hundred µl of bead solution was seeded on a 3.5 cm glass bottom dish and added to 0.5 units of thrombin (Sigma). After 15 minutes incubation at 37°C in 5% CO_2_ incubator, fibroblasts (WI-38 cells, 400,000 cells/ml) in 1 ml of EGM-2 MV media were harvested onto fibrin gels. After 3–7 days incubation, sprouting HUVECs were observed using spinning disk microscopy.

### Time-lapse imaging of zebrafish embryos

All experiments using zebrafish were approved by the IACUC of Yale University. Zebrafish (Danio rerio) embryos were raised as previously described [68]. The Tg(kdrl:ras-mCherry) [69] transgenic line was used for injection. The Tol2 system was used for the injection of the plasmid for transient expression in embryos [70]. Briefly, the zebrafish VEC gene was cloned from zebrafish cDNA and cloned into pDestTol2pA2, the transposon-donor plasmid with a fli1 promoter sequence and SV40 polyA signal. About 1 nl of a DNA/RNA solution, containing 25 ng/ul transposon-donor plasmid and 25 ng/ul transposase mRNA in 10 mM HEPES buffer, were co-injected into one-cell stage embryos using microinjection. The injected embryos were raised at 28°C in egg water with 0.002% 1-phenyl-2-thiourea to prevent pigment development. Embryos were placed for time lapse analysis in 3.5 cm glass bottom dishes in egg water containing 1% low melting point agarose with 0.016% tricaine to inhibit movement of the embryos. Zebrafish VEC-EGFP expression in endothelial cells of embryos was analyzed at 30–80 h post fertilization (hpf) using spinning disk microscopy. The embryos maintained heartbeat and circulation throughout the imaging period.

### Statistical analysis

All experiments were performed independently at least three times. Data were expressed as mean ± standard deviation (SD). Comparison between groups was performed with a two-tailed Student's t test. Results were considered significant at *P*<0.01.

## Supporting Information

Figure S1 VEC-EGFP expressed in COS7 cells forms adherens junctions and is internalized by adjacent cells. (**A**) COS7 cells expressing VEC-EGFP were fixed and stained with anti-N-cadherin antibody. Exogenously expressed VEC formed adherens junctions and excluded N-cadherin from junctions in COS7 cells. Scale bar  = 20 µm. (**B**) Time-lapse imaging of co-culture of COS7 cells expressing VEC-EGFP and COS7 cells expressing VEC-TagRFPT. Arrowheads show internalized VEC-TagRFPT molecules by adjacent VEC-EGFP expressing cells. The number of internalized VEC-TagRFPT molecules in VEC-EGFP expressing cells increased gradually in a time-dependent manner. Scale bar  = 10 µm. (**C**) Quantitative analysis of the number of trans-endocytosed VEC-TagRFPT molecules in B. The number of trans-endocytosed molecules was counted for over 3 different fields of view per time point. N = 3. (**D and E**) Co-culture of HUVECs expressing VEC-EGFP and HUVECs expressing VEC-mKikGR. Lower images are higher magnification of the indicated area in upper images. Before photo-activation, almost no fluorescence was detected in the red channel. After photo-activation, trans-endocytosed VEC-mKikGR molecules were detected in VEC-EGFP positive cells. Arrowheads show trans-endocytosed VEC-mKikGR molecules by VEC-EGFP expressing cells. Scale bar  =  upper images, 20 µm; 5 µm, lower images. (**F**) The number of trans-endocytosis positive cells was counted over time after mixing of HUVECs expressing VEC-EGFP and HUVECs expressing VEC-mKikGR. Arrowheads show internalized VEC-mKikGR molecules by VEC-EGFP expressing cells. The number of trans-endocytosis positive cells increased in a time-dependent manner. Scale bar  = 40 µm. (**G**) Quantitative analysis of the number of trans-endocytosis positive cells shown in F. The numbers of trans-endocytosis positive cells were counted over 6-9 different fields of view for each time point; n = 37 (2 h), n = 50 (4 h), n = 55 (6 h), n = 42 (8 h) and n = 32 (10 h). (**C and G**) Data were expressed as mean ± SD. *, p<0.01, vs control cells by ANOVA, Tukey HSD Test.
(TIFF)

Figure S2 Trans-endocytosis requires formation of cell-cell junctions. (**A**) Co-culture of HUVECs expressing VEC-EGFP (EGFP cell) and HUVECs expressing VEC-TagRFPT (TagRFPT cell) using Transwell plates, which allow medium exchange between two cell lines. Serial observations after plating showed no indication of the trans-endocytosis. Scale bar  = 10 µm. (**B**) The exosomal fraction in the culture medium. We confirmed that the exosomal fraction, while positive for the exosomal marker Syntenin, did not contain VEC. For the marker for non-exosomal fraction, anti-calnexin (the marker for the endoplasmic reticulum) antibody was used. (**C**) Co-culture of HUVECs expressing VEC-EGFP and HUVECs expressing VEC-W49A-TagRFPT. VEC mutant (VEC-W49A-TagRFPT) did not interact with VEC of adjacent cells. When cell-cell junction formation was disrupted by VEC-W49A-TagRFPT, the trans-endocytosis of VEC did not occur. Scale bar  = 10 µm.
(TIFF)

Figure S3 p120- or β-catenin-EGFP and VEC-TagRFPT are trans-endocytosed by the neighboring cells concurrently. (**A**) Co-culture of COS7 cells expressing both β-catenin-EGFP and VEC-TagRFPT and iRFP expressing HUVECs. β-catenin-EGFP and VEC-TagRFPT were trans-endocytosed by neighboring cells concurrently. Scale bar  = 20 µm. (**B**) Co-culture of COS7 cells expressing both p120-catenin-EGFP and VEC- TagRFPT and iRFP expressing HUVECs. p120-EGFP and VEC-TagRFPT were trans-endocytosed by neighboring cells concurrently. Scale bar  = 20 µm.
(TIFF)

Figure S4 VEC trans-endocytosis is not dependent on clathrin-dependent endocytosis nor macropinocytosis. (**A**) Co-culture of HUVECs expressing VEC-EGFP and HUVECs expressing VEC-TagRFPT with fluorescently labeled transferrin. The trans-endocytosed VEC molecules by an adjacent cell showed no co-localization with endocytosed transferrin. Lower images are higher magnification of the indicated area in upper images. Scale bars  = 10 µm, upper images; 5 µm, lower images. (**B**) Co-culture of HUVECs expressing VEC-EGFP and HUVECs expressing mRFP-Rab5 or mRFP-Rab5-DN. Arrows show trans-endocytosed VEC-EGFP molecules by mRFP-Rab5 and mRFP-Rab5-DN expressing cells. Scale bar  = 10 µm. (**C**) Co-culture of HUVECs expressing VEC-EGFP and HUVECs expressing VEC-TagRFPT with or without siRNAs against macropinocytosis markers. Arrowheads show trans-endocytosed VEC-TagRFPT molecules by VEC-EGFP expressing cells. The VEC trans-endocytosis occurred even with siRNAs against macropinocytic markers. Lower images are higher magnification of the indicated area in upper images. Scale bars  = 20 µm, upper images; 5 µm, lower images.
(TIFF)

Figure S5 Co-localization of trans-endocytosed molecules with Rab proteins in the receiving cells. (**A**) Co-culture of HUVECs expressing VEC-TagRFPT and either EGFP-Rab5, EGFP-Rab7 or EGFP-Rab11. The internalized VEC molecule from the neighboring cell co-localized with a subset of Rab7-positive endosomes and a small subset of Rab5- and Rab11-positive endosomes, in the receiving cells. (**B**) Quantification of the number of trans-internalized vesicles co-localized with Rab proteins in receiving cells. The numbers of co-localized vesicles in the cells were counted over 11–14 different fields of view for each Rab proteins; n = 14 (EGFP-Rab5), n = 14 (EGFP-Rab7) and n = 11 (EGFP-Rab11).
(TIFF)

Figure S6 Rac1 inhibition suppresses VE-cadherin trans-endocytosis in a dose-dependent manner. (**A**) Co-culture of HUVECs expressing VEC-EGFP and HUVECs expressing iRFP with W56. W56 is the peptide of the GEF recognition/activation site of Rac1 and acts as a Rac1 inhibitor. IC_50_ of W56 is 100 µM. VEC trans-endocytosis was inhibited by W56 in a dose-dependent and time-dependent manner. (**B and C**) Quantitative analysis of immuno-staining in A. The number of trans-endocytosis positive cells was counted over 11–13 different fields of view for each point; n = 31–39 (W56, 1 h) and n = 28–50 (W56, o/n). *, p<0.01 vs DMSO. Data were expressed as mean ± SD.
(TIFF)

Movie S1 Time-lapse imaging of co-culture of COS7 cells expressing VEC-EGFP and HUVECs expressing VEC-TagRFP657. The interaction between endogenous VEC in HUVECs and over-expressed VEC-EGFP in COS7 cells can induce trans-endocytosis. The trans-endocytosed VEC-EGFP molecules from a COS7 cell appeared to bud off from cell-cell junctions and be pulled into a HUVEC. The time-lapse images were acquired with a 60x objective lens every 13 seconds for 25 minutes by spinning disk confocal microscopy and were played back as movie at 10 frames per second.
(MOV)

Movie S2 Time-lapse imaging of co-culture of HUVECs expressing VEC-EGFP and HUVECs expressing LifeAct-TagRFPT. The internalized VEC-EGFP vesicle clearly associated with F-actin visualized by LifeAct-TagRFPT. The time-lapse images were acquired with a 60x objective lens every 1 minute for 42 minutes by spinning disk confocal microscopy and were played back as movie at 10 frames per second.
(MOV)

Movie S3 Time-lapse imaging of co-culture of HUVECs expressing VEC-EGFP and HUVECs expressing VEC-TagRFPT with (−)-blebbistatin. 100 µM of (−)-blebbistatin was added at 20 minutes during the time-lapse acquisition and inhibited trans-endocytosis of VEC. The time-lapse images were acquired with a 60x objective lens every 80 seconds for 60 minutes by spinning disk confocal microscopy and were played back as movie at 4 frames per second.
(MOV)

Movie S4 Time-lapse imaging of co-culture of HUVECs expressing VEC-EGFP and HUVECs expressing PA-Rac1-CA or PA-Rac1-DN. Upper images are time-lapse imaging of co-culture of HUVECs expressing VEC-EGFP and HUVECs expressing PA-Rac1-CA. PA-Rac1-CA accumulated at cell-cell junction and co-localized with VEC-EGFP after its activation. Lower images are time-lapse imaging of co-culture of HUVECs expressing VEC-EGFP and HUVECs expressing PA-Rac1-DN. PA-Rac1-DN did not accumulate at cell-cell junction, nor did it co-localize with VEC-EGFP after its activation. The time-lapse images were acquired with a 60x objective lens every 36 seconds for 29 minutes by spinning disk confocal microscopy and were played back as movie at 7 frames per second.
(MOV)

Movie S5 Time-lapse imaging of sprouting HUVECs in the three-dimensional fibrin gel culture. Some vesicles were trans-endocytosed from VEC-EGFP positive HUVECs into VEC-TagRFPT positive HUVECs in the three-dimensional fibrin gel culture. The time-lapse images were acquired with a 60x objective lens every minute for 32 minutes by spinning disk confocal microscopy and were played back as movie at 10 frames per second.
(MOV)

Movie S6 Time-lapse imaging of live zebrafish embryo expressing VEC-EGFP. Endothelial cells expressing VEC-EGFP at intersegmental vessel of a zebrafish embryo were observed for 17 minutes. Arrows showed a zVEC-EGFP positive vesicle budding into inside of the endothelial cell. The time-lapse images were acquired with a 60x objective lens every 1 minute for 17 minutes by spinning disk confocal microscopy and were played back as movie at 5 frames per second.
(MOV)
