# Supplementary figures and images for: Inter-Cellular Exchange of Cellular Components via VE-Cadherin-Dependent Trans-Endocytosis
Source: PLoS One. 2014 Mar 6;9(3):e90736. doi: 10.1371/journal.pone.0090736 (PMC3946293; doi:10.1371/journal.pone.0090736)

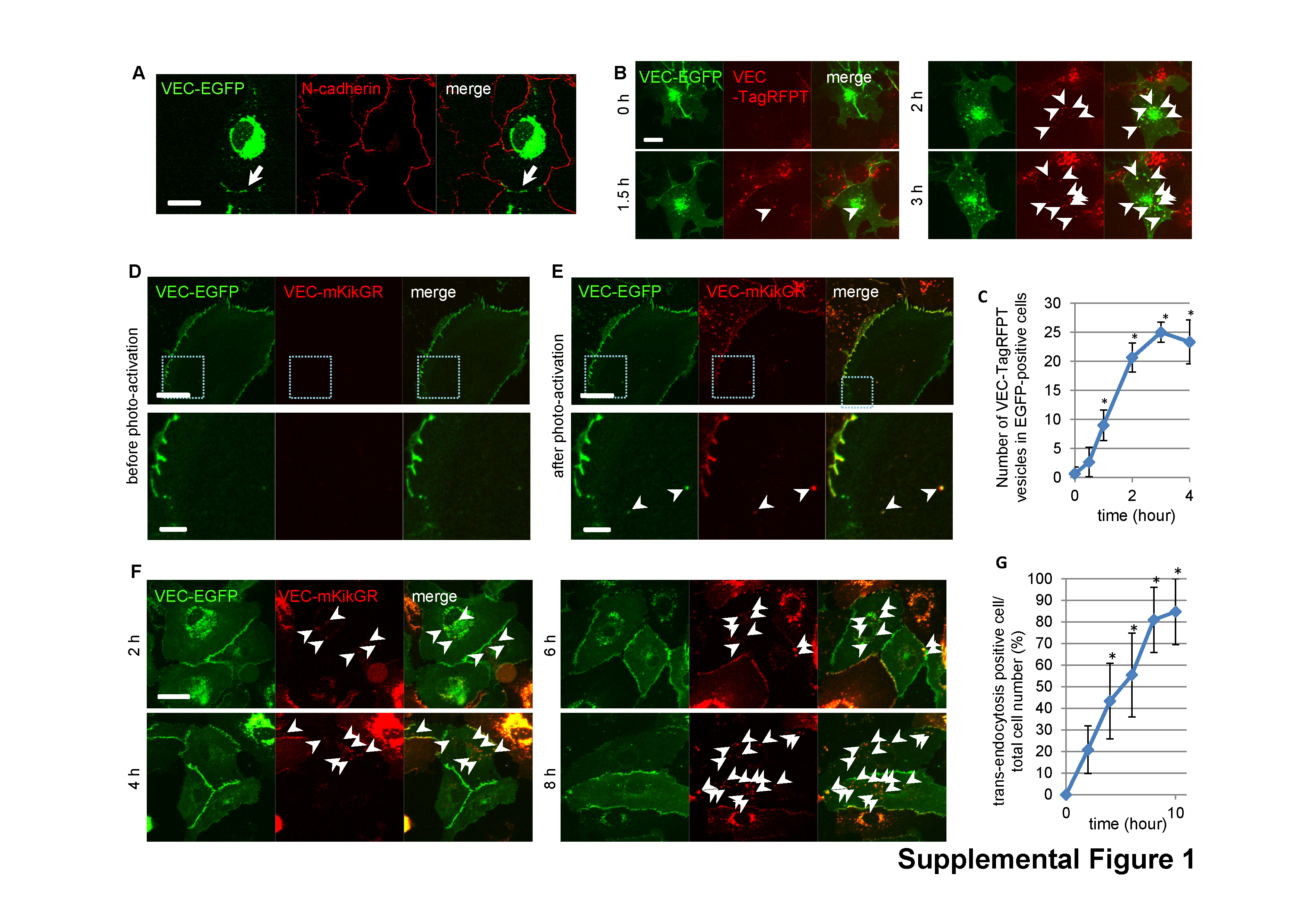

Supplement: Figure S1 — VEC-EGFP expressed in COS7 cells forms adherens junctions and is internalized by adjacent cells. (A) COS7 cells expressing VEC-EGFP were fixed and stained with anti-N-cadherin antibody. Exogenously expressed VEC formed adherens junctions and excluded N-cadherin from junctions in COS7 cells. Scale bar = 20 µm. (B) Time-lapse imaging of co-culture of COS7 cells expressing VEC-EGFP and COS7 cells expressing VEC-TagRFPT. Arrowheads show internalized VEC-TagRFPT molecules by adjacent VEC-EGFP expressing cells. The number of internalized VEC-TagRFPT molecules in VEC-EGFP expressing cells increased gradually in a time-dependent manner. Scale bar = 10 µm. (C) Quantitative analysis of the number of trans-endocytosed VEC-TagRFPT molecules in B. The number of trans-endocytosed molecules was counted for over 3 different fields of view per time point. N = 3. (D and E) Co-culture of HUVECs expressing VEC-EGFP and HUVECs expressing VEC-mKikGR. Lower images are higher magnification of the indicated area in upper images. Before photo-activation, almost no fluorescence was detected in the red channel. After photo-activation, trans-endocytosed VEC-mKikGR molecules were detected in VEC-EGFP positive cells. Arrowheads show trans-endocytosed VEC-mKikGR molecules by VEC-EGFP expressing cells. Scale bar = upper images, 20 µm; 5 µm, lower images. (F) The number of trans-endocytosis positive cells was counted over time after mixing of HUVECs expressing VEC-EGFP and HUVECs expressing VEC-mKikGR. Arrowheads show internalized VEC-mKikGR molecules by VEC-EGFP expressing cells. The number of trans-endocytosis positive cells increased in a time-dependent manner. Scale bar = 40 µm. (G) Quantitative analysis of the number of trans-endocytosis positive cells shown in F. The numbers of trans-endocytosis positive cells were counted over 6-9 different fields of view for each time point; n = 37 (2 h), n = 50 (4 h), n = 55 (6 h), n = 42 (8 h) and n = 32 (10 h). (C and G) Data were expressed as mean ± SD. [file pone.0090736.s001.tif]

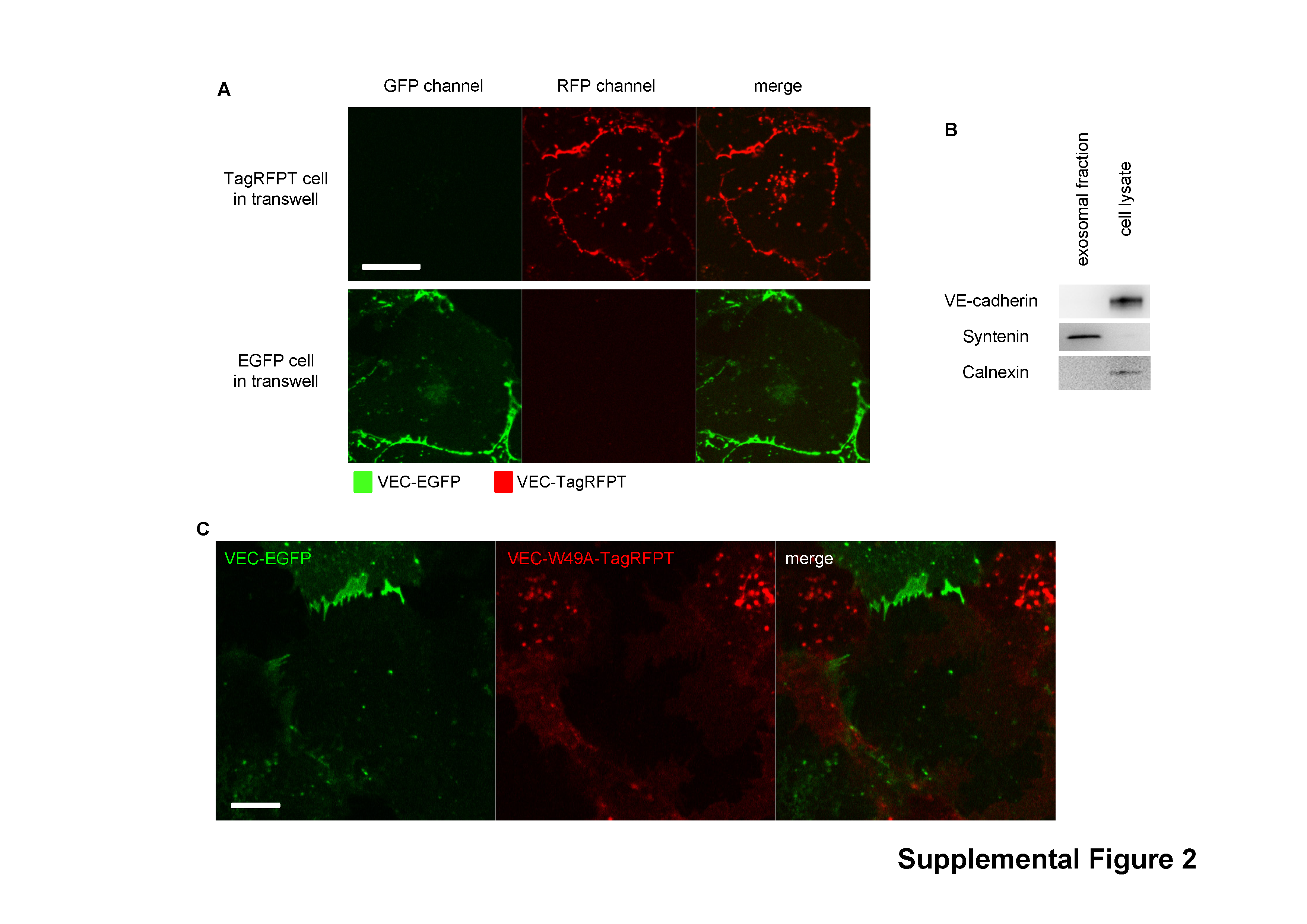

Supplement: Figure S2 — Trans-endocytosis requires formation of cell-cell junctions. (A) Co-culture of HUVECs expressing VEC-EGFP (EGFP cell) and HUVECs expressing VEC-TagRFPT (TagRFPT cell) using Transwell plates, which allow medium exchange between two cell lines. Serial observations after plating showed no indication of the trans-endocytosis. Scale bar = 10 µm. (B) The exosomal fraction in the culture medium. We confirmed that the exosomal fraction, while positive for the exosomal marker Syntenin, did not contain VEC. For the marker for non-exosomal fraction, anti-calnexin (the marker for the endoplasmic reticulum) antibody was used. (C) Co-culture of HUVECs expressing VEC-EGFP and HUVECs expressing VEC-W49A-TagRFPT. VEC mutant (VEC-W49A-TagRFPT) did not interact with VEC of adjacent cells. When cell-cell junction formation was disrupted by VEC-W49A-TagRFPT, the trans-endocytosis of VEC did not occur. Scale bar = 10 µm. (TIFF) [file pone.0090736.s002.tif]

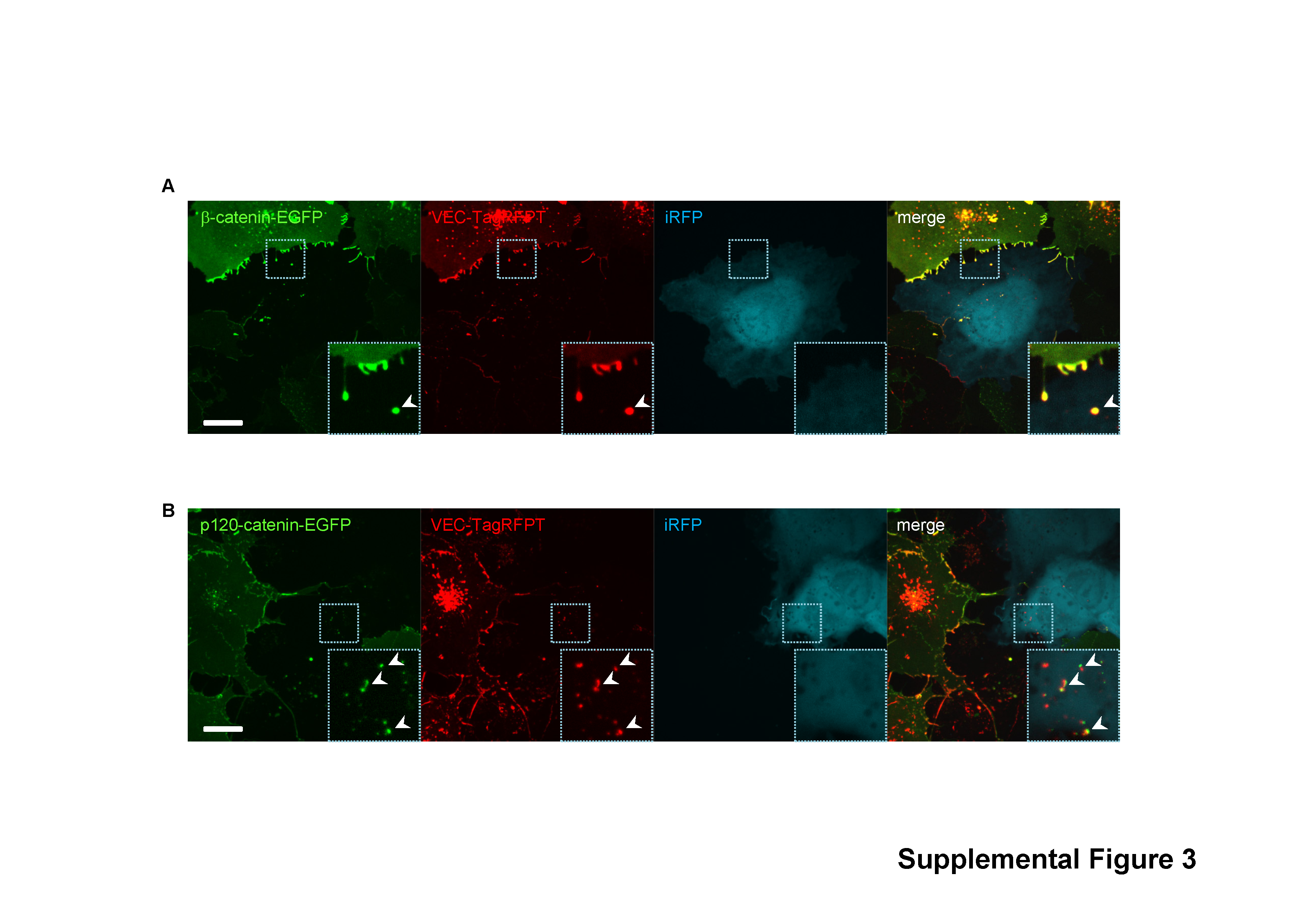

Supplement: Figure S3 — p120- or β-catenin-EGFP and VEC-TagRFPT are trans-endocytosed by the neighboring cells concurrently. (A) Co-culture of COS7 cells expressing both β-catenin-EGFP and VEC-TagRFPT and iRFP expressing HUVECs. β-catenin-EGFP and VEC-TagRFPT were trans-endocytosed by neighboring cells concurrently. Scale bar = 20 µm. (B) Co-culture of COS7 cells expressing both p120-catenin-EGFP and VEC- TagRFPT and iRFP expressing HUVECs. p120-EGFP and VEC-TagRFPT were trans-endocytosed by neighboring cells concurrently. Scale bar = 20 µm. (TIFF) [file pone.0090736.s003.tif]

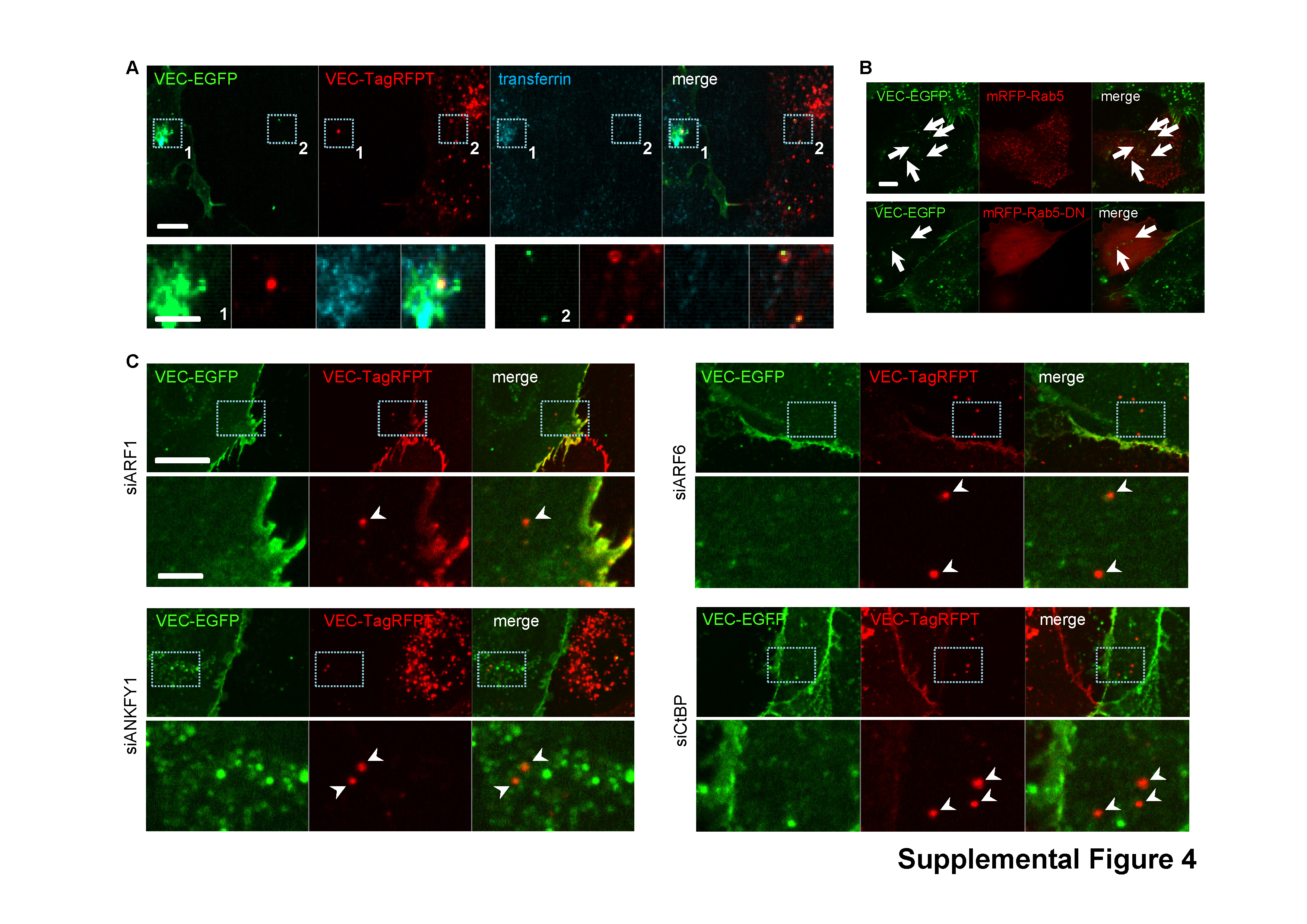

Supplement: Figure S4 — VEC trans-endocytosis is not dependent on clathrin-dependent endocytosis nor macropinocytosis. (A) Co-culture of HUVECs expressing VEC-EGFP and HUVECs expressing VEC-TagRFPT with fluorescently labeled transferrin. The trans-endocytosed VEC molecules by an adjacent cell showed no co-localization with endocytosed transferrin. Lower images are higher magnification of the indicated area in upper images. Scale bars = 10 µm, upper images; 5 µm, lower images. (B) Co-culture of HUVECs expressing VEC-EGFP and HUVECs expressing mRFP-Rab5 or mRFP-Rab5-DN. Arrows show trans-endocytosed VEC-EGFP molecules by mRFP-Rab5 and mRFP-Rab5-DN expressing cells. Scale bar = 10 µm. (C) Co-culture of HUVECs expressing VEC-EGFP and HUVECs expressing VEC-TagRFPT with or without siRNAs against macropinocytosis markers. Arrowheads show trans-endocytosed VEC-TagRFPT molecules by VEC-EGFP expressing cells. The VEC trans-endocytosis occurred even with siRNAs against macropinocytic markers. Lower images are higher magnification of the indicated area in upper images. Scale bars = 20 µm, upper images; 5 µm, lower images. (TIFF) [file pone.0090736.s004.tif]

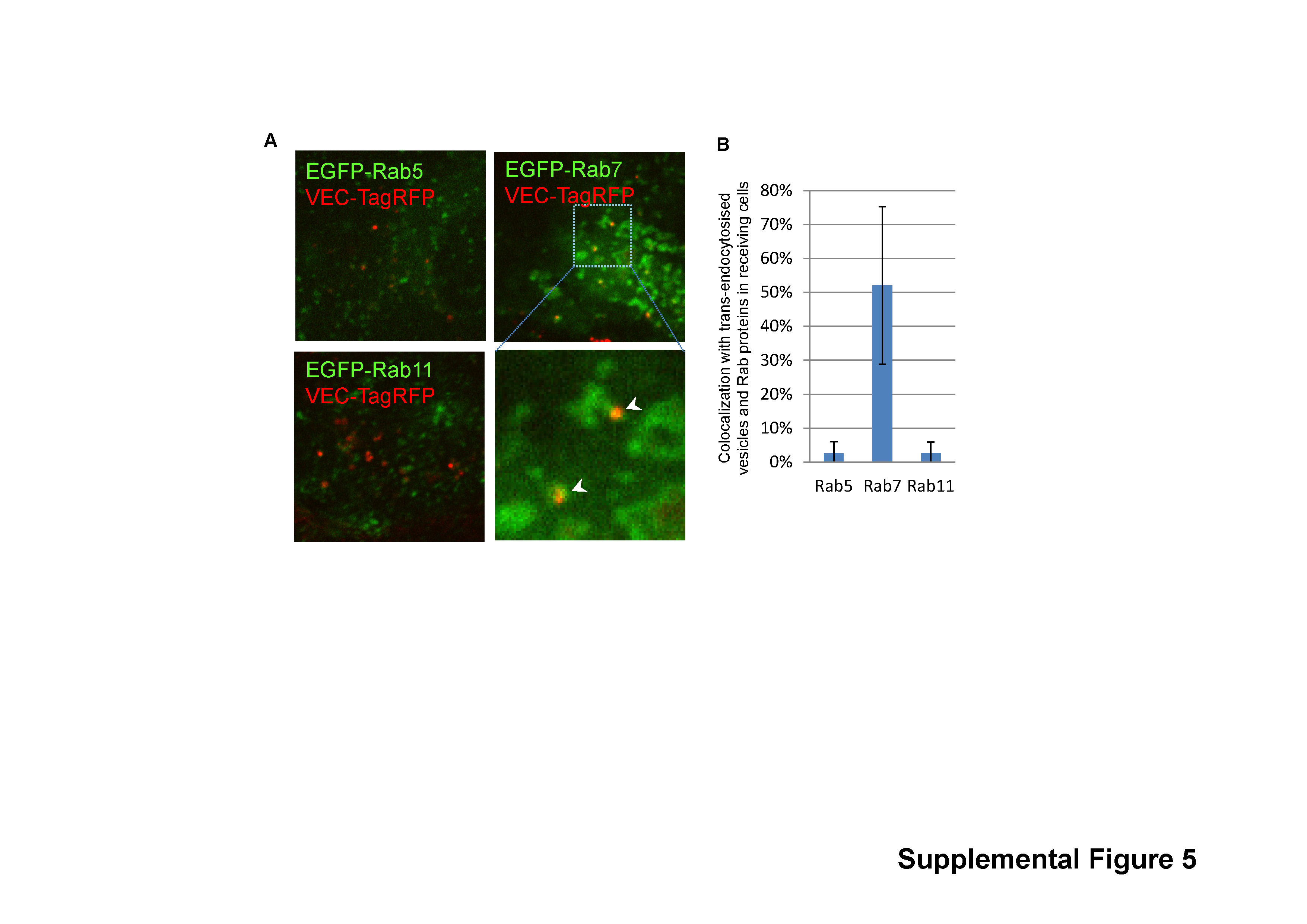

Supplement: Figure S5 — Co-localization of trans-endocytosed molecules with Rab proteins in the receiving cells. (A) Co-culture of HUVECs expressing VEC-TagRFPT and either EGFP-Rab5, EGFP-Rab7 or EGFP-Rab11. The internalized VEC molecule from the neighboring cell co-localized with a subset of Rab7-positive endosomes and a small subset of Rab5- and Rab11-positive endosomes, in the receiving cells. (B) Quantification of the number of trans-internalized vesicles co-localized with Rab proteins in receiving cells. The numbers of co-localized vesicles in the cells were counted over 11–14 different fields of view for each Rab proteins; n = 14 (EGFP-Rab5), n = 14 (EGFP-Rab7) and n = 11 (EGFP-Rab11). (TIFF) [file pone.0090736.s005.tif]

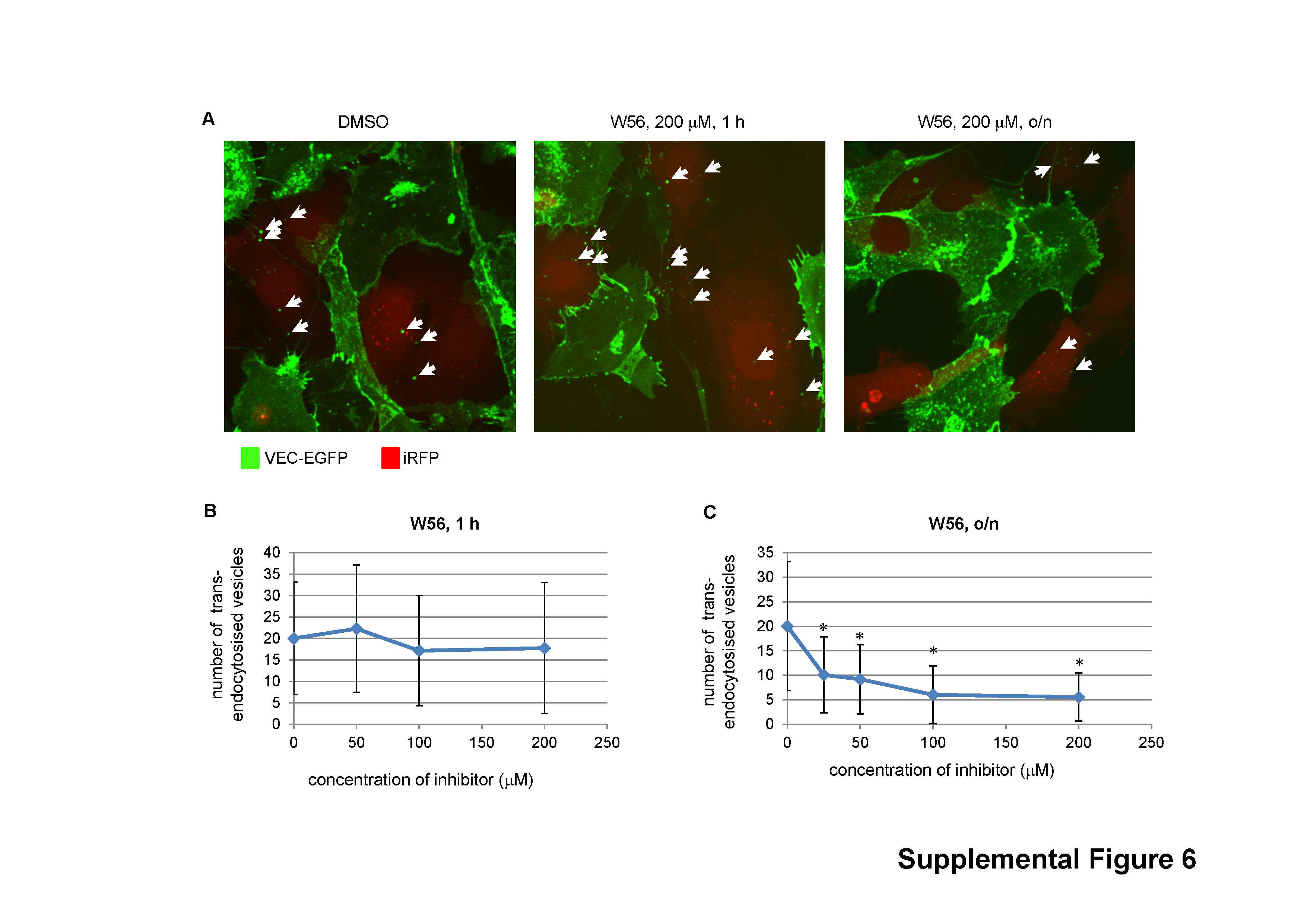

Supplement: Figure S6 — Rac1 inhibition suppresses VE-cadherin trans-endocytosis in a dose-dependent manner. (A) Co-culture of HUVECs expressing VEC-EGFP and HUVECs expressing iRFP with W56. W56 is the peptide of the GEF recognition/activation site of Rac1 and acts as a Rac1 inhibitor. IC50 of W56 is 100 µM. VEC trans-endocytosis was inhibited by W56 in a dose-dependent and time-dependent manner. (B and C) Quantitative analysis of immuno-staining in A. The number of trans-endocytosis positive cells was counted over 11–13 different fields of view for each point; n = 31–39 (W56, 1 h) and n = 28–50 (W56, o/n). *, p<0.01 vs DMSO. Data were expressed as mean ± SD. (TIFF) [file pone.0090736.s006.tif]
